# Dying among older adults in Switzerland: who dies in hospital, who dies in a nursing home?

**DOI:** 10.1186/s12904-016-0156-x

**Published:** 2016-09-23

**Authors:** Xhyljeta Luta, Radoslaw Panczak, Maud Maessen, Matthias Egger, David C. Goodman, Marcel Zwahlen, Andreas E. Stuck, Kerri Clough - Gorr

**Affiliations:** 1 Institute of Social and Preventive Medicine, University of Bern, Finkeubelweg 11, CH-3012 Bern, Switzerland; 2 University Department of Geriatrics, Inselspital Bern, Bern, Switzerland; 3 Section of Geriatrics, Boston University Medical Center, Boston, MA USA; 4 The Dartmouth Institute for Health Policy & Clinical Practice, Lebanon, New Hampshire USA

**Keywords:** Institutional deaths, End of life, Variation, Hospital service areas, Small area analysis, Switzerland

## Abstract

**Background:**

Institutional deaths (hospitals and nursing homes) are an important issue because they are often at odds with patient preference and associated with high healthcare costs. The aim of this study was to examine deaths in institutions and the role of individual, regional, and healthcare supply characteristics in explaining variation across Swiss Hospital Service Areas (HSAs).

**Methods:**

Retrospective study of individuals ≥66 years old who died in a Swiss institution (hospital or nursing homes) in 2010. Using a two-level logistic regression analysis we examined the amount of variation across HSAs adjusting for individual, regional and healthcare supply measures. The outcome was place of death, defined as death in hospital or nursing homes.

**Results:**

In 2010, 41,275 individuals ≥66 years old died in a Swiss institution; 54 % in nursing homes and 46 % in hospitals. The probability of dying in hospital decreased with increasing age. The OR was 0.07 (95 % CI: 0.05–0.07) for age 91+ years compared to those 66–70 years. Living in peri-urban areas (OR = 1.06 95 % CI: 1.00–1.11) and French speaking region (OR = 1.43 95 % CI: 1.22–1.65) was associated with higher probability of hospital death. Females had lower probability of death in hospital (OR = 0.54 95 % CI: 0.51–0.56). The density of ambulatory care physicians (OR = 0.81 95 % CI: 0.67–0.97) and nursing homes beds (OR = 0.67 95 % CI: 0.56–0.79) was negatively associated with hospital death. The proportion of dying in hospital varied from 38 % in HSAs with lowest proportion of hospital deaths to 60 % in HSAs with highest proportion of hospital deaths (1.6-fold variation).

**Conclusions:**

We found evidence for variation across regions in Switzerland in dying in hospital versus nursing homes, indicating possible overuse and underuse of end of life (EOL) services.

**Electronic supplementary material:**

The online version of this article (doi:10.1186/s12904-016-0156-x) contains supplementary material, which is available to authorized users.

## Background

Dying in an institutional setting (hospital or nursing home) is an important issue in end of life (EOL) care because it is often not aligned with patient preferences and is associated with high healthcare costs. An important goal of EOL care is to enable people to die free of pain and in the place of their choice [1, 2]. Studies on preferences for place of death have demonstrated that home is where most people want to die [3, 4]. A systematic review including 175 studies from 33 countries found that the majority of people prefer to die at home. The preference for death at home among patients ranged between 31 and 87 % [5]. Dying at home has been associated with availability of home-based palliative care services, disease (cancer versus other diagnosis), early transfer to end of life care, and presence of caregiver [6].

Care provided just before death varies by type of institution. Compared to nursing homes, inpatient acute care hospitals (hereafter referred to as hospitals) provide more intensive and costly EOL care [7]. Despite being considered as “inappropriate” setting for dying patients, hospital care may be needed in circumstances where clinical needs of the patient cannot be met in other settings [8]. Hall et al. [9] suggest that other factors including transfers to other settings may be associated with less hospital deaths. Moreover, patients may prefer the hospital setting due to fear of death, or because they believe that hospitals provide better care [10]. However, there is some indication of changing trends in place of death in the past years [3]. Wilson et al. (2014) examined factors associated with shifts in deaths outside hospital in Canada after 1994 which appear to be related to socio-demographic developments, changes in the health care systems as well as improvement in EOL services over the years [11].

Where a person dies varies by geographic location [6]. Other factors related to place of death include differences in health status of the population across regions and characteristics of the health care system (e.g. supply of health care, health care staff characteristics) [12, 13].

As in other developed countries, the Swiss population is ageing. It is predicted that by 2060 the percentage of people aged 65 and older will increase to 28 % of the population [14]. This demographic change will lead to new healthcare system challenges in caring for large numbers of older adults including EOL care. Despite the associated high costs, few studies have examined institutional deaths in Switzerland [15, 16]. Information on regional variation of institutional deaths and the causes of this variation is also limited. Although healthcare insurance is uniform for the Swiss population, healthcare organization varies across the country. Within a fragmented healthcare system, such as that in Switzerland, it is essential for physicians and health policy makers to understand variations in order to improve EOL care.

The aim of this study was to describe regional differences in the proportion of institutional deaths across 71 hospital services areas (HSAs) in Switzerland. We included all deaths that occurred in institutions in 2010 in Switzerland. We compared deaths that occurred in hospital versus nursing homes. Using a multilevel analysis we examined regional variation in institutional deaths adjusting for individual (age, sex), regional (e.g. urbanicity, language region) and healthcare supply measures (e.g. density of physicians, density of hospital beds). We hypothesized that these factors partly explain differences across regions in place of death in Switzerland.

## Methods

### Conceptual framework

To guide our analysis, we developed a conceptual framework of selected factors related to variation in place of death in Switzerland (Additional file 1). Many factors influencing place of death have been reported in literature [13, 17]. In our conceptual framework, we hypothesize that variation in institutional deaths in Switzerland may occur as a result of interplay between three levels of determinants: (a) macro level determinants (e.g. health policy); (b) meso level determinants (e.g. healthcare supply); (c) micro level determinants (e.g. individual level characteristics). Due to unavailable data we were not able to include all variables presented in the model. The included variables are described in detail below.

### Study setting

The Swiss federation is divided into 26 cantons and three main language regions: German (63.5 %) French (22.5 %) Italian (8.1 %) [18]. The majority of population lives in urban areas (75 %), of this 35 % live in five main cities (Bern, Basel, Geneva, Lausanne and Zurich) [19] (Additional file 2).

### Study design and participants

We conducted a retrospective analysis of individuals who died in a Swiss institution in 2010 and were 66 years or older at the time of death. We restricted to age 66 and over because underlying causes of death, preferences and EOL experiences of children and younger adults are different than older adults and because majority of deaths occur in the older population. Patients with missing or incorrect place of residence were excluded because they could not be assigned to HSAs (*N* = 521). We excluded patients from prisons because this group of was too small to constitute a separate category and likely to have different characteristics (*N* = 6).

**Fig. 1:**
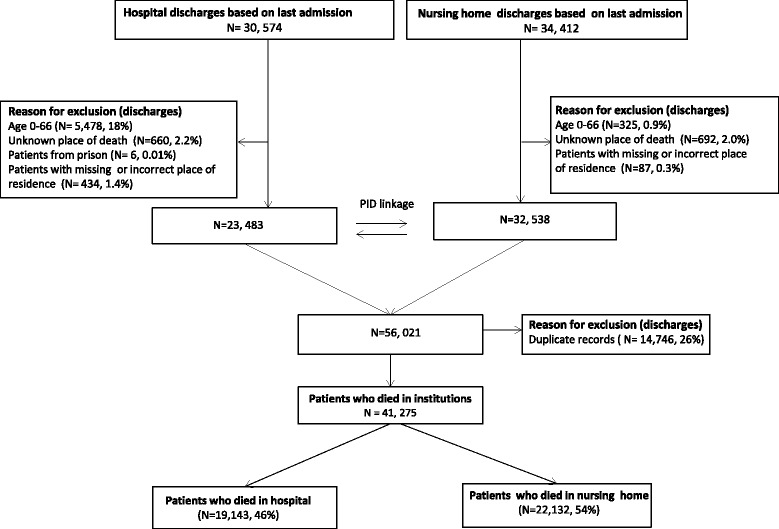
Flowchart of study population with exclusion criteria

We further excluded records with unknown place of death (*N* = 1,352). Detailed information on selection of the study population is provided in Fig. 1. The study was approved by the cantonal ethics committee of Bern.

### Outcome measure

The outcome measure is place of death, which we defined either as death in hospital or death in nursing home.

### Data sources

We obtained data from five sources: micro level patient characteristics from Hospital Discharges (HD), Socio-medical Institutions (SOMED), Federal Death Statistics (FDS), Classification of Communities (CC) all from the Swiss Federal Statistical Office (SFSO), and meso level characteristics from the Swiss Medical Association (SMA) and Hospital Infrastructure (HI) dataset (SFSO). The SOMED and HD data were fully anonymized and linked using a common person identifier (PID). A detailed description of variables unique to each dataset is provided below.

### Analytic variables

#### Individual characteristics

Data included information on age at time of death aggregated by six age bands (66–70, 71–75, 76–80, 81–85, 86–90 and 91+ years), sex and disease (main diagnosis at hospital admission based on International Classification of Diseases, 10th revision, ICD-10). Due to lack of ICD-10 codes among people who died in nursing homes, we could only use ICD-10 codes of people who died in hospital. Information on hospital admission characteristics included: type of admission, location before admission, referring institution, department of inpatient care and insurance-based room category.

#### Regional characteristics

Regional characteristics were derived from CC dataset. Regional variables included language region (German, French, and Italian), and urbanicity (urban, peri-urban, and rural) measured on 564 modified Medstat regions. Socioeconomic position of region of residence (SEP) was based on tertiles (1^st^ highest to 3^rd^ lowest) of the Swiss neighborhood index of SEP. Detailed information describing the development of the Swiss-SEP has been published elsewhere [20].

#### Healthcare supply measures

Inpatient supply measures were obtained from HI data. This included information on density of in-patient physicians, density of overall beds, acute beds and nursing homes. According to SFSO [21], there are five types of non-acute institutions in Switzerland: homes for the elderly, nursing homes, and institutions for people with disabilities, institutions for people with addictions and institutions for people with psychosocial problems. For the purpose of this study, we included only nursing homes. Finally, we used SMA data to describe characteristics of ambulatory services. This includes information on general practitioners (GP) and specialists. Healthcare supply measures were calculated per 10,000 inhabitants and divided into tertiles for analytic purposes.

### Hospital service areas

Described in detail elsewhere [22], a HSA is a defined geographic area with at least one hospital where most patients receive care, irrespective of political boundaries. In short, we created HSAs using a complete database of 2010 discharges and regions of place of residence of hospitalization (Medstat). A matrix of patient flow counts was generated and used to aggregate Medstat regions to the regions with highest number of hospitalizations. In this study, 71 geographically distinct HSAs were defined.

### Statistical analysis

We used descriptive statistics to provide a general overview of the study population. The main analysis included unadjusted and adjusted two-level mixed logistic regression models with place of death (hospital versus nursing homes) as outcome. Results from multilevel analysis are reported as odds ratio (OR) with 95 % confidence intervals (95 % Cl) and estimates of between HSA variance with standard error (SE). The data had a two-level hierarchical structure with individuals (level 1) nested within 71 HSAs (level 2). Model 1, the unadjusted model, contained only an intercept separating the variance on the individual level and HSA level. Model 2 included individual level variables (age, gender). Model 3 included individual and regional characteristics (language region, urbanicity, Swiss-SEP). Model 4, included all variables in Model 3 plus healthcare supply measures (density of physicians, total hospital beds, acute care beds, nursing homes beds and ambulatory care physicians).

As a sub - analysis we examined main diagnosis at last hospital admission and admission characteristics of hospital deaths. In a sensitivity analysis, we stratified models by gender. All analyses were done in Stata version 14 (StataCorp, College Station, Texas, USA).

## Results

A total of 52, 037 deaths occurred in 2010 among individuals aged 66 or older (Additional file 3); of these 41, 275 died in institutions, which equates to 79, 3 % of deaths among patients aged 66 and older in older in 2010. We assume that the remaining deaths (21 %) occurred at home or outside home due to accidents or violence. Table 1 provides details on characteristics of patients according to the type of institution. Deaths occurred more frequently in nursing homes (54 %) than in hospital (46.4 %). Nursing homes deaths were more common than hospital deaths in persons aged between 86 and 90 (≤64 % vs. ≥36 %), and age 91 and over (≤79.2 % vs. ≥21 %). Females died more often in nursing homes than in hospital (≤63 % vs. ≥37.2). The distribution of institutional deaths also varied by regional characteristics. The highest percentage of hospital deaths occurred in French speaking part of Switzerland (53.4 %) compared to Italian and German speaking part. There were more deaths in nursing homes in German speaking part of Switzerland (56.4 %). Peri-urban areas had the highest percentage of hospital deaths (49.4 %) compared to urban and rural areas, whereas more deaths in nursing homes occurred in rural areas (57 %).

**Table 1:** Individual and regional characteristics by place of death of individuals aged 66 or older who died in 2010

Characteristics	Hospital			Nursing home			Total institutions	
	N	Column %	Row%	N	Column %	Row %	N	Column %
Sex								
Male	10,310	54.0	59.0	7,247	33.0	41.3	17,557	42.5
Female	8,833	46.1	37.2	14,885	67.3	63.0	23,718	57.5
Age								
66–70	2,353	12.3	82.0	520	2.3	18.1	2,873	7.0
71–75	2,815	15.0	76.0	899	4.1	24.2	3,714	9.0
76–80	3,814	20.0	65.0	2,082	9.4	35.3	5,896	14.3
81–85	4,427	23.1	50.2	4,389	20.0	50.0	8,816	21.4
86–90	3,736	19.5	36.0	6,639	30.0	64.0	10,375	25.1
91+	1,998	10.4	21.0	7,603	34.4	79.2	9,601	23.3
Language region^a^								
German	12,786	67.0	44.0	16,529	75.0	56.4	29,315	71.0
French	5,173	27.0	53.4	4,516	20.4	47.0	9,689	23.5
Italian	1,184	6.2	52.1	1,087	5.0	48.0	2,271	5.5
Urbanicity^a^								
Urban	6,621	35.0	45.1	8,045	36.3	55.0	14,666	35.5
Peri-urban	8,183	43.0	49.4	8,372	38.0	50.6	16,555	40.1
Rural	4,339	23.0	43.2	5,715	26.0	57.0	10,054	24.4
Swiss-SEP index^a^								
1st (lowest)	4,019	21.0	46.0	4,767	21.5	54.3	8,786	21.3
2nd tertile	7,944	41.5	46.5	9,148	41.3	53.5	17,092	41.4
3rd (highest)	7,180	37.5	47.0	8,217	37.1	53.4	15,397	37.3
Total	19,143	100.0	46.4	22,132	100.0	54.0	41,275	100.0

^a^Language region, urbanicity and Swiss-SEP index are measured at Medstat level

### Multilevel analysis

Results of multilevel analysis are shown in Table 2. Results of Model 1 showed substantial variation in institutional deaths between HSAs. In Model 2, the addition of age and sex increased the variance from 012 to 0.14. There was a strong association between age and the likelihood of dying in hospital. Compared to age 66–70 years the probability of dying in hospital decreased with increasing age. For example, the OR was 0.07 (95 % CI: 0.06–0.07) for age 91 or older (Table 2). Females were less likely to die in hospital than males (OR = 0.54, 95 % CI: 0.51–0.56).

**Table 2:** Odds ratios and 95 % confidence intervals comparing the probability of death in hospital with nursing homes. Results from multilevel models with individual, regional and healthcare supply variables (*N* = 41, 275)

Characteristics	Model 1	Model 2 OR [95 % CI]	Model 3 OR [95 % CI]	Model 4 OR [95 % CI]
Age in years				
66–70	--	Reference	Reference	Reference
71–75	--	0.70 [0.62,0.80]	0.71 [0.62,0.79]	0.71 [0.62,0.79]
76–80	--	0.42 [0.37,0.47]	0.42 [0.37,0.46]	0.42 [0.37,0.46]
81–85	--	0.24 [0.21,0.26]	0.24 [0.21,0.26]	0.24 [0.21,0.26]
86–90	--	0.14 [0.12,0.15]	0.14 [0.12,0.15]	0.14 [0.12,0.15]
91+	--	0.07 [0.06,0.07]	0.07 [0.05,0.07]	0.07 [0.05,0.07]
Sex				
Male	--	Reference	Reference	Reference
Female	--	0.54 [0.51,0.56]	0.54 [0.51,0.56]	0.54 [0.51,0.56]
Language region				
German	--		Reference	Reference
French	--		1.55 [1.32,1.80]	1.43 [1.22,1.65]
Italian	--		1.85 [1.16,2.92]	1.80 [1.20,2.70]
Urbanicity				
Urban	--		Reference	Reference
Peri-urban	--		1.06 [1.00,1.11]	1.06 [1.00,1.11]
Rural	--		0.95 [0.87,1.03]	0.95 [0.87,1.02]
Swiss-SEP index				
1st (lowest)	--		Reference	Reference
2nd tertile	--		1.08 [0.99,1.17]	1.08 [0.98,1.17]
3rd (highest)	--		1.05 [0.93,1.16]	1.04 [0.93,1.16]
Hospital beds (overall)/10,000				
1st (lowest)	--			Reference
2nd tertile	--			0.97 [0.80,1.16]
3rd (highest)	--			0.95 [0.77,1.15]
Acute care beds per/10,000				
1st (lowest)	--			Reference
2nd tertile	--			1.10 [0.92,1.31]
3rd (highest)	--			1.14 [0.93,1.39]
Physicians (inpatient)/10,000				
1st (lowest)	--			Reference
2nd tertile	--			0.98 [0.81,1.18]
3rd (highest)	--			1.07 [0.85,1.33]
Ambulatory care (GPs & specialists)/10,000				
1st (lowest)	--			Reference
2nd tertile	--			0.84 [0.70,1.01]
3rd (highest)	--			0.81 [0.67,0.97]
Nursing home beds /10,000				
1st (lowest)	--			Reference
2nd tertile	--			0.83 [0.70,0.98]
3rd (highest)	--			0.67 [0.56,0.79]
Variance				
Estimate	0.12	0.14	0.08	0.05
SE	0.02	0.02	0.01	0.01

* *OR Odds ratio, *Cl* Confidence Interval, *SE* Standard error, language region, urbanicity, SEP (Medstat level), health care supply measures (HSA level). Ambulatory care physician are measured at Medstat level. All other supply measures at HSA level

In Model 3, the addition of regional variables reduced between-HSA variance by 33 % (from 0.12 to 0.08). Results regarding age and sex were nearly identical. Living in peri-urban areas was associated with higher probability of dying in hospital (OR = 1.06, 95 % CI: 1.00–1.11) compared to urban and rural settings. The probability of death in hospital was higher in the French and Italian speaking regions than in the German speaking region (OR = 1.55, 95 % CI: 1.32–1.80 and OR = 1.85, 95 % CI: 1.16–2.92, respectively). There was no association between dying in hospital and SEP of region of place of residence. For example, the OR in the highest tertile was 1.05 (95 % CI: 0.93–1.16).

In Model 4, the addition of healthcare supply measures further reduced the between-HSAs variance by 37 % (from 0.08 to 0.05). The density of ambulatory care physicians in the region was negatively associated with death in hospital (OR = 0.81, 95 % CI: 0.67–0.97), comparing the highest with the lowest tertile. Similarly, the density of nursing homes was negatively associated with death hospital (OR = 0.67, 95 % CI: 0.56–0.79), comparing the highest with the lowest tertile. We did not find an association between dying in hospital and the density of total hospital beds (OR = 0.95, 95 % CI: 0.77–01.15), acute care beds (OR = 1.14, 95 % CI: 0.93–1.39) and inpatient physicians (OR = 1.07, 95 % CI: 0.85–1.33), comparing highest with the lowest tertile.

**Fig. 2:**
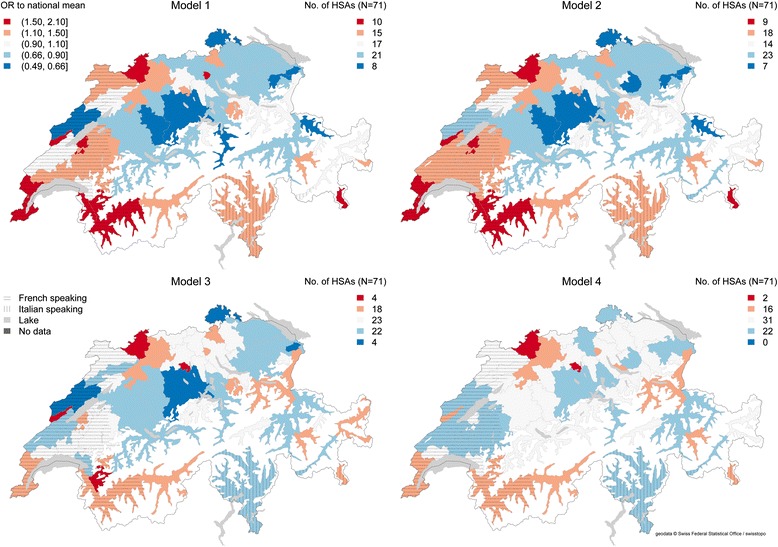
Odds of dying in hospital versus nursing homes across 71 HSAs among patients 66 or older. Model 1 (null model). Model 2 (adjusted for age and sex). Model 3 (adjusted for individual and regional characteristics). Model 4 (adjusted for individual, regional and healthcare supply measures). Dark red indicates HSAs with highest odds of dying in hospital

Figure 2 shows maps of odds of dying in hospital compared to nursing homes based on the unadjusted model (Model 1), adjusted for sex and age (Model 2), adjusted for individual and regional characteristics (Model 3) and the fully adjusted model (Model 4).

Figure 2 shows the results of Model 1 or null model. The HSAs with the highest odds of hospital deaths were located in the French speaking part of Switzerland (northern part of canton of Vaud, Geneva and Valais) and in a small HSA located in the Italian speaking part (southwestern part of canton of Graubünden).

In Model 4 a considerable variation was reduced compared to Model 1 and Model 2 after including regional characteristics and healthcare supply measures. The likelihood of dying in hospital was higher in greater Basel region and northern part of the canton of Solothurn.

**Fig. 3:**
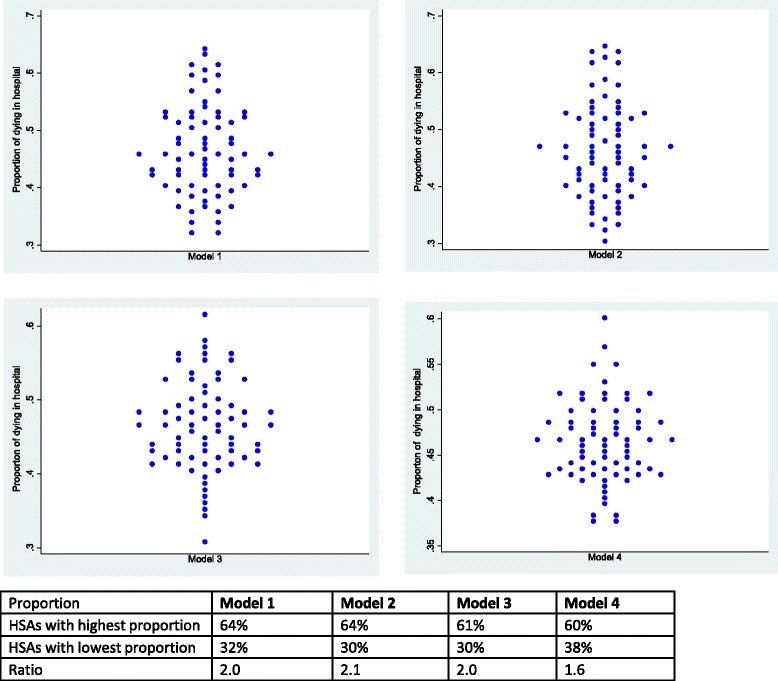
Proportion of dying in hospital versus nursing homes across 71 HSAs among patients 66 or older. Model 1 (null model). Model 2 (adjusted for age and sex). Model 3 (adjusted for individual and regional characteristics). Model 4 (adjusted for individual, regional and healthcare supply measures). Each point represents one of the 71 HSAs in Switzerland. The tables beneath each figure show the highest and lowest proportion as well as ratio

Figure 3 shows predicted proportions for dying in hospital compared to dying in nursing homes based on the unadjusted model (Model 1), adjusted for age and sex (Model 2), adjusted for individual and regional characteristics (Model 3) and the fully adjusted model (Model 4). In Model 1 and Model 2, death in hospital varied by a factor of 2 among HSAs, from 32 to 64 % and 30 to 64 % respectively. In Model 3, death in hospital varied by a factor of 2 from 30 to 61 %. In Model 4, death in hospital varied by a factor of 1.6 from 38 % in HSAs with lowest proportion to 60 % in HSAs with highest proportion.

In a sub-analysis, we found that the leading main diagnosis at the last admission among hospital deaths was neoplasms (23.5 %), disease of circulatory system (27 %), and diseases of respiratory system (10.3 %) (Additional file 4). The majority of patients who died in hospitals were admitted in an emergency department (72.3 %) on the last admission, were at home before admission (73.1 %), were referred to hospital by a physician (54.%), received care in internal medicine departments (65.4 %) and were hospitalized in shared rooms (77.%) (Additional file 5). In a sensitivity analysis, after stratifying models by gender, we found several differences between males and females in a fully adjusted model. Compared to males, we found no association between living in peri-urban and dying in hospital among females (OR = 1.03, 95 % CI: 0.96–1.10). Females living in the Italian speaking part of Switzerland were more likely to die in hospital than males (OR = 1.97, 95 % CI: 1.28–3.01).We found no association among males between the density of ambulatory care physicians (OR = 0.88, 95 % CI: 0.72–1.09) and dying in hospital.

## Discussion

### Summary of main findings

In this multilevel analysis, we found substantial variation across HSAs with regard to dying in hospitals versus nursing homes among people 66 years and older in Switzerland. The results show that dying in institutions in Switzerland is not only a function of individual factors. Patients living in French speaking part were more likely to die in hospital. Living in peri-urban areas was associated with higher probability of dying in hospital compared to urban and rural settings. There was also an association between institutional death and several healthcare supply measures. Our findings indicate that multiple factors at both the micro and meso levels influence dying in institutions in Switzerland.

### Comparison with previous literature

Our findings are in line with previous research showing that a high proportion of patients die in hospitals [19]. Similar findings were reported in Germany (39.3 %) [23]. The proportion of hospital deaths, however, appears to be higher in Australia, (54 %) [24] Czech Republic (58.4 %), Slovakia (54.8 %) [25], Canada (51 %) [26] and United Kingdom (58 %) [27] than in Switzerland (36.7 %). A study comparing hospital deaths in six European countries found huge differences in proportion of hospital deaths across countries ranging from 33.9 % in Netherlands to 62.8 % in Wales [28]. Such differences were only partially explained by the availability of healthcare supply. Pivodic et al. [12] examined factors associated with place of death across 14 countries. Similar to our findings, the authors found a considerable variation in place of death which could be partly explained by individual and healthcare supply characteristics.

According to a 2009 Swiss survey [24], 73 % of the participants preferred dying at home over institutional settings. With nearly 80 % of deaths occurring in institutions, it is likely that many patients in Switzerland are not dying in the setting they prefer. Others have shown that preferences for the place of death may change over time [5, 25]. However, for many EOL patients, hospital settings might be necessary because of complications that cannot be easily managed in other settings [8]. Furthermore, death in hospital may be unexpected. Different diseases have different disease trajectories and death can be difficult to predict [26]. Previous studies have reported variation in place of death across diseases [27, 28]. Knowing cause of death, unavailable for this study, could inform institutional death analyses.

We found that individual patient characteristics were associated with whether patients died in hospital or nursing homes. Consistent with previous research, we also found that people who die in hospital are more likely to be males and younger, [29, 30] whereas females live longer than their male counterparts and in EOL are more likely to live and die in nursing homes [31]. In our study, females were more likely to die in nursing homes irrespective of age. We report a slightly higher probability of death in nursing homes in peri-urban areas. One EOL study has shown that nursing homes use is higher in areas with increased availability of such facilities [6] although this analysis was not focused on place of death. A recently published study [32] indicates that 99 % of population in Switzerland can reach at least one nursing homes facility within 15 minutes. Similar to a 2009 American study, we did not find a significant association between SEP and institutional deaths [33]. However, overall published findings on the topic are mixed with some studies observing that higher socio-economic groups are less likely to die in hospital compared to those with lower SEP [34, 35].

Some of the regional variation in this study may reflect cultural differences across regions in Switzerland [36]. Higginson et al. (2014) found out that patients with immigrant background were significantly more likely to die in a hospital setting than their UK counterparts [37]. We also report an association of institutional deaths with the three different language regions in Switzerland; these are generally considered to be a proxy for cultural regions. These regions may reflect different understandings and beliefs about EOL, as well as different local practice patterns [38].

We found that deaths in nursing homes were more likely in areas where the density of nursing homes beds was greater. Similar results were reported in a USA study [17]. Another explanation could be the availability of community services across regions. For example, in eastern Switzerland, people are more likely to use nursing homes compared to the western part where there is a higher use of home care (SPITEX) [39]. We also report that HSAs with higher density of GPs and specialists had lower probability of dying in hospital. This is consistent with previous research showing the important role of GPs in reducing high hospitalisation rates at EOL [40]. However, in our study, density of hospital beds was not associated with hospital death, and contradicts previous reports showing an increased likelihood of death in hospital in regions with higher supply of hospital beds. However, the confidence interval for acute care beds was wide and the trend is in the expected direction. Previous studies have demonstrated [33, 41] an increased likelihood of death in hospital in regions with higher supply of hospital beds, in our study, density of hospital beds was not associated with dying hospital.

### Limitations and strengths

We acknowledge several limitations in our study. First, our study is based on retrospective data collected for administrative purposes [42]. Reuse of such data is efficient, but is accompanied by incomplete clinical information and inaccuracies in coding [43]. Data on preferences were not available. Therefore, it was not possible to determine whether patients died in their preferred setting. EOL care is directly related to disease and as previously mentioned this information was not available for nursing home patients. Despite these limitations, our study has several strengths. To our knowledge, this is the first study in Switzerland which examines regional variation in institutional deaths across HSAs. Another strength is the large sample size. Finally, hierarchical models allowed us to simultaneously analyse the effects of ecological-level and individual-level variables [44].

### Implications for EOL care

These findings have potentially important implications for patients, clinicians and policy makers alike. They can be used to inform Swiss policy towards addressing regional differences for reducing EOL costs and ensuring EOL care in line with patient preferences. HSAs with high proportion of hospital deaths might indicate overuse or inappropriate use of costly acute care hospital resources (e.g. patients living in nursing homes might be inappropriately admitted to acute care hospital). HSAs with low proportion of hospital deaths might indicate an underuse of needed acute care hospital services at EOL (e.g. a nursing homes resident is not offered needed acute care hospital admission). Therefore, an in-depth analysis with information on quality of care is needed and is a high priority.

This work should also encourage future research that can account for patient preferences, cause of death and changes over time. These Swiss findings have relevance for other developed countries because of similar demographic and healthcare delivery problems effecting EOL care (e.g. aging, rising healthcare costs, concerns that patient preferences are not always followed). The unexplained variation in our analysis could be due to unmeasured factors (e.g. patient preferences, local practice patterns, underlying population health) [45]. Some variation may be expected as it may reflect differences in population need, and cultural characteristics of the population or patient preferences, which does not necessarily imply unwarranted variation [46]. The focus should be on unwarranted variation or variation that cannot be explained by patient differences but by healthcare capacity amenable to change [47].

## Conclusions

We found evidence for variation in institutional deaths across HSAs which can partly be explained by individual, regional and healthcare supply characteristics. HSAs should be further explored for overuse and underuse of EOL services. Further efforts are needed to examine the potential causes of these variations using additional data on individual-level (e.g. preferences), healthcare system (e.g. type of EOL services) as well as data to analyse trends of institutional deaths over time.

## Additional files

Additional file 1:
**Figure S1.** Conceptual framework of selected aspects influencing death in institution. (PDF 61 kb) Additional file 2:
**Figure S2.** Map of Swiss geography. (PDF 701 kb) Additional file 3:
**Table S1.** Individual and regional characteristics of study population including deaths in institutions and total deaths in Switzerland in 2010 among patients aged 66 and older. (DOCX 21 kb) Additional file 4:
**Table S2.** Main diagnosis at last admission based on International Classification of Diseases, 10th revision, ICD-10. (DOC 32 kb) (DOCX 17 kb) Additional file 5:
**Table S3.** Admission characteristics of people who died in hospital based on last admission. (DOCX 15 kb)

## Abbreviations

CI: Classification of communities
EOL: End of life
FDS: Federal Death Statistics
HD: Hospital discharges
HI: Hospital infrastructure
HSA: Hospital service area
ICD-10: International Statistical Classification of Diseases and Related Health Problems
SFSO: Swiss Federal Statistical Office
SMA: Swiss Medical Association
SOMED: Socio-Medical Institutions
