# Supplementary figures and images for: Dying among older adults in Switzerland: who dies in hospital, who dies in a nursing home?
Source: BMC Palliat Care. 2016 Sep 23;15:83. doi: 10.1186/s12904-016-0156-x (PMC5035491; doi:10.1186/s12904-016-0156-x)

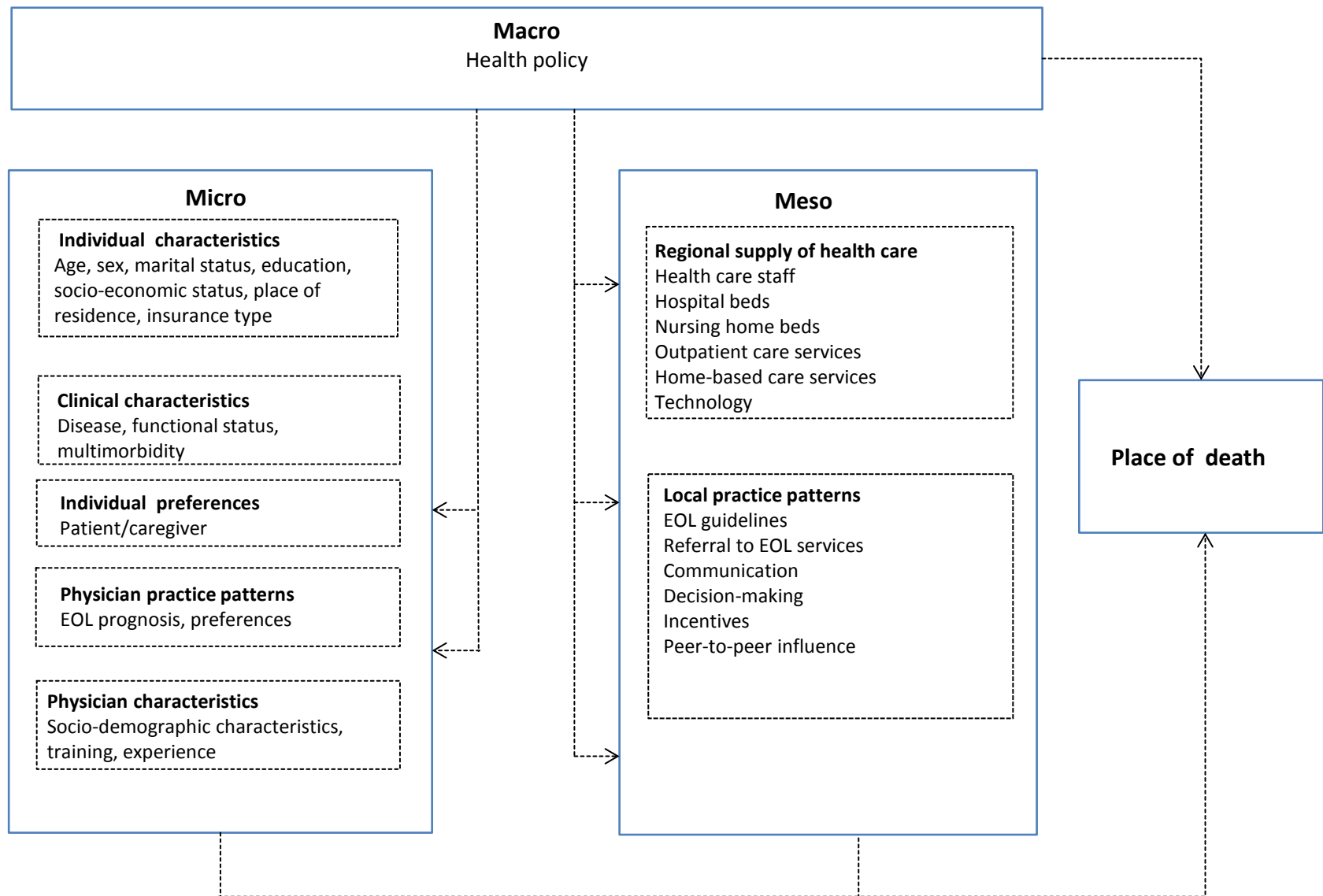

**Figure S1.** Conceptual framework of selected aspects influencing place of death.

Supplement: Additional file 1: — Figure S1. Conceptual framework of selected aspects influencing death in institution. (PDF 61 kb) [file 12904_2016_156_MOESM1_ESM.pdf]
