# Supplementary material for: Dying among older adults in Switzerland: who dies in hospital, who dies in a nursing home?
Source: BMC Palliat Care. 2016 Sep 23;15:83. doi: 10.1186/s12904-016-0156-x (PMC5035491; doi:10.1186/s12904-016-0156-x)
Supplement: Additional file 2: — Figure S2. Map of Swiss geography. (PDF 701 kb) [file 12904_2016_156_MOESM2_ESM.pdf]

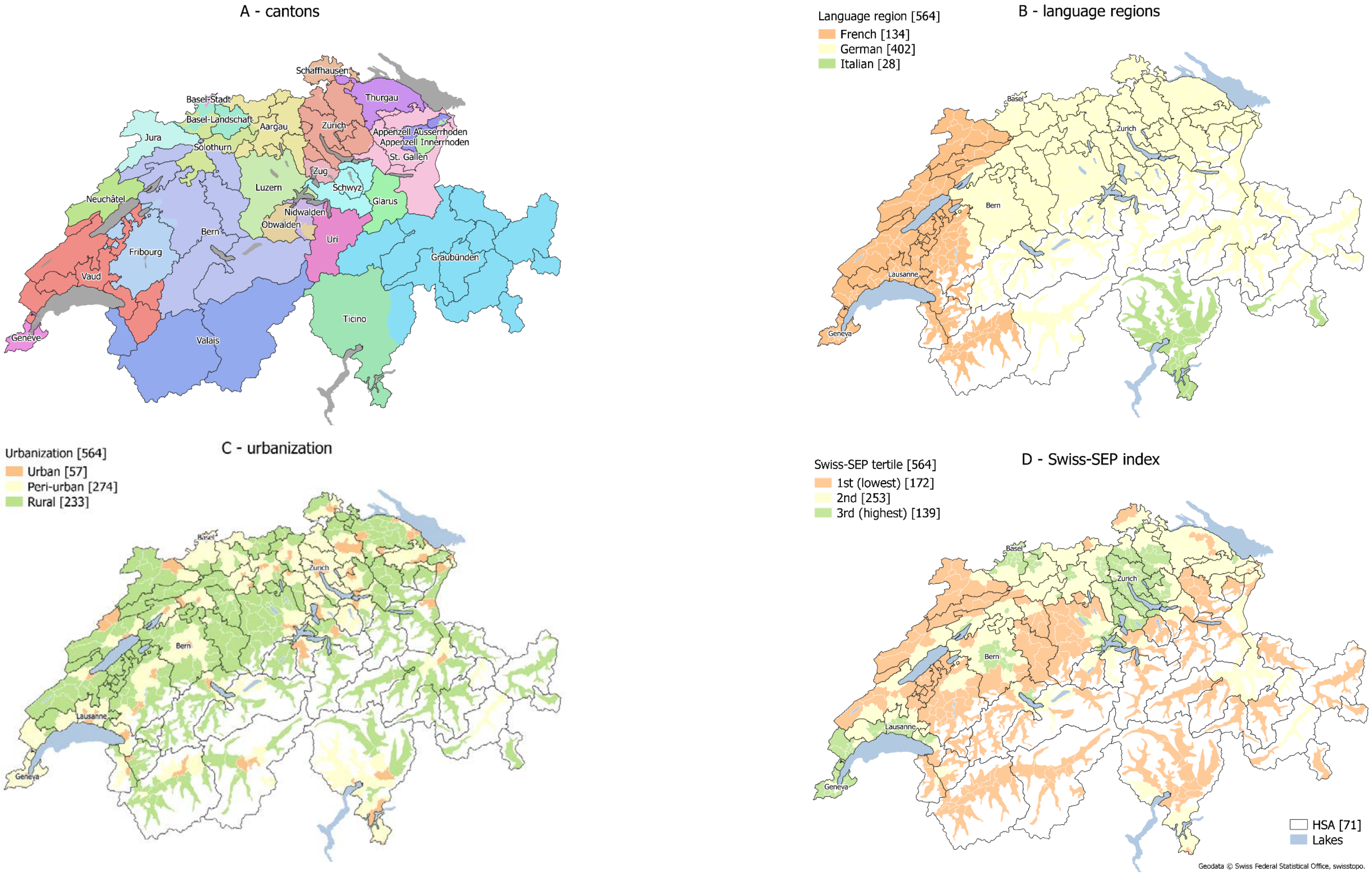

A - cantons
Schaffhausen
Basel-Stadt
Thurgau
Basel-Landschaft
Aargau
Zürich
Jura
Solothurn
Appenzell Ausserrhoden
Appenzell Innerrhoden
St. Gallen
Zug
Schwyz
Luzern
Neuchâtel
Glarus
Nidwalden
Obwalden
Bern
Uri
Fribourg
Graubünden
Vaud
Ticino
Genève
Valais
B - language regions
Language region [564]
French [134]
German [402]
Italian [28]
Basel
Zurich
Bern
Lausanne
Geneva
C - urbanization
Urbanization [564]
Urban [57]
Peri-urban [274]
Rural [233]
Basel
Zurich
Bern
Lausanne
Geneva
D - Swiss-SEP index
Swiss-SEP tertile [564]
1st (lowest) [172]
2nd [253]
3rd (highest) [139]
Basel
Zurich
Bern
Lausanne
Geneva
HSA [71]
Lakes
Geodata © Swiss Federal Statistical Office, swisstopo.
