# Supplementary material for: Dying among older adults in Switzerland: who dies in hospital, who dies in a nursing home?
Source: BMC Palliat Care. 2016 Sep 23;15:83. doi: 10.1186/s12904-016-0156-x (PMC5035491; doi:10.1186/s12904-016-0156-x)
Supplement: Additional file 3: — Table S1. Individual and regional characteristics of study population including deaths in institutions and total deaths in Switzerland in 2010 among patients aged 66 and older. (DOCX 21 kb) [file 12904_2016_156_MOESM3_ESM.docx]

| **Characteristics** | **Institutions** | | **Total deaths in 2010** | | | | |
| --- | --- | --- | --- | --- | --- | --- | --- |
|  | N | Column % | N | Column % | | | |
| **Sex** |  |  |  | | |  | |
| Males | 17,557 | 42.5 | 23,637 | | 45.4 | |  |
| Females | 23,718 | 57.5 | 28,400 | | 55.0 | |  |
| **Age** |  |  |  | |  | |  |
| 66 - 70 | 2,873 | 7.0 | 4,191 | | 8.1 | |  |
| 71 - 75 | 3,714 | 9.0 | 5,326 | | 10.2 | |  |
| 76 - 80 | 5,896 | 14.3 | 8,047 | | 15.5 | |  |
| 81 - 85 | 8,816 | 21.4 | 11,276 | | 22.0 | |  |
| 86 - 90 | 10,375 | 25.1 | 12,489 | | 24.0 | |  |
| 91+ | 9,601 | 23.3 | 10,708 | | 20.6 | |  |
| **Language region** |  |  |  | |  | |  |
| German | 29,315 | 71.0 | 37,497 | | 72.1 | |  |
| French | 9,689 | 23.5 | 11,883 | | 23.0 | |  |
| Italian | 2,271 | 5.5 | 2,657 | | 5.1 | |  |
| **Urbanicity** |  |  |  | |  | |  |
| Urban | 14,666 | 35.5 | 17,965 | | 34.5 | |  |
| Peri-urban | 16,555 | 40.1 | 21,129 | | 41.0 | |  |
| Rural | 10,054 | 24.4 | 12,943 | | 25.0 | |  |
| **Swiss-SEP index** |  |  |  | |  | |  |
| 1st(lowest) | 8,786 | 21.3 | 11,148 | | 21.4 | |  |
| 2nd | 17,092 | 41.4 | 21,401 | | 41.1 | |  |
| 3rd (highest) | 15,397 | 37.3 | 19,488 | | 37.5 | |  |
| Total | 41,275 | 100.0 | 52,037 | | 100.0 | |  |

*Language region, urbanicity and Swiss-SEP index are measured at Medstat level.
