# Supplementary material for: Dying among older adults in Switzerland: who dies in hospital, who dies in a nursing home?
Source: BMC Palliat Care. 2016 Sep 23;15:83. doi: 10.1186/s12904-016-0156-x (PMC5035491; doi:10.1186/s12904-016-0156-x)
Supplement: Additional file 4: — Table S2. Main diagnosis at last admission based on International Classification of Diseases, 10th revision, ICD-10. (DOC 32 kb) (DOCX 17 kb) [file 12904_2016_156_MOESM4_ESM.docx]

| **ICD-10** | **Hospital deaths** |
| --- | --- |
| **Age 66 or older** | N ( %) |
| Neoplasms (C00-D48) | 4,494 (23.5) |
| Circulatory system (I00-I99) | 5,177 (27.0) |
| Respiratory system (J00-J99) | 1,980 (10.3) |
| Injury, posioning & consequences of external causes (S00-T98) | 1,494 (7.8) |
| Digestive system (K00-K93) | 1,502 (7.8) |
| Infectious and parasitic diseases (A00-B99) | 1,459 (7.6) |
| Factors influencing health status & contact with health services (Z00-Z99) | 505 (2.6) |
| Genitourinary system (N00-N99) | 520 (2.7) |
| Nervous system (G00-G99) | 362 (1.9) |
| Mental and behavioural disorders (F00-F99) | 288 (1.5) |
| Other | 1,345 (7.0) |
| Missing | 17 (0.1) |
| Total | 19,143 (100.0) |

**Note:** Only patients who died in hospital are included in this table.
