# Supplementary material for: Dying among older adults in Switzerland: who dies in hospital, who dies in a nursing home?
Source: BMC Palliat Care. 2016 Sep 23;15:83. doi: 10.1186/s12904-016-0156-x (PMC5035491; doi:10.1186/s12904-016-0156-x)
Supplement: Additional file 5: — Table S3. Admission characteristics of people who died in hospital based on last admission. (DOCX 15 kb) [file 12904_2016_156_MOESM5_ESM.docx]

| **Admission characteristics** | **Hospital deaths N ( %)** |
| --- | --- |
| **Type of admission** |  |
| Emergency services | 13,846 (72.3) |
| Planned | 4,880 (25. 5) |
| Other | 399 (2.1) |
| Unknown | 18 (0.1) |
|  |  |
| **Location before admission** |  |
| Home | 13,989 (73.1) |
| Non-acute institutions | 1,165 (6.1) |
| Other hospitals | 3,453 (18.0) |
| Other | 467 (2.4) |
| Unknown | 69 (0.4) |
|  |  |
| **Referral instance** |  |
| Patient/Relatives | 1,980 (10.3) |
| Emergency | 6,325 (33.0) |
| Physician | 10,343 (54.0) |
| Other | 310 (1.6) |
| Unknown | 185 (1.0) |
|  |  |
| **Main cost type** |  |
| General medicine | 323 (1.7) |
| Intensive care | 457 (2.4) |
| Internal medicine | 12,515 (65.4) |
| Surgery | 2,886 (15.1) |
| Radiology | 51 (0.3) |
| Geriatrics | 1,659 (9.0) |
| Other | 1,252 (6.5) |
|  |  |
| **Room category (insurance-based)** |  |
| Shared | 14,654 (77.0) |
| Semi-private | 2,889 (15.1) |
| Private | 1,589 (8.3) |
| Unknown | 11 (0.1) |
| Total | 19,143 (100.0) |

**Note:** Only patients who died in hospital are included in this table.
